# Mediastinal malignant mesothelioma discovered in a patient with dysphagia

**DOI:** 10.1002/rcr2.592

**Published:** 2020-06-08

**Authors:** Dario Amore, Simona Massa, Umberto Caterino, Dino Casazza, Albina Palma, Carlo Curcio

**Affiliations:** ^1^ Department of Thoracic Surgery Monaldi Hospital Naples Italy; ^2^ Complex Operative Unit of Pathology Monaldi Hospital Naples Italy; ^3^ Thoracic Endoscopic Unit Monaldi Hospital Naples Italy; ^4^ Emergency Department C.T.O Naples Italy

**Keywords:** Mediastinal mass, mesothelioma, thoracic surgery

## Abstract

A mediastinal mass in patients with a history of asbestos exposure should raise the suspicion of malignant mesothelioma.

## Graphical abstract

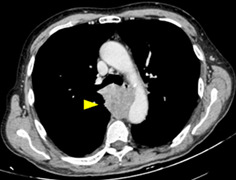

## Clinical Image

**Figure 1:**
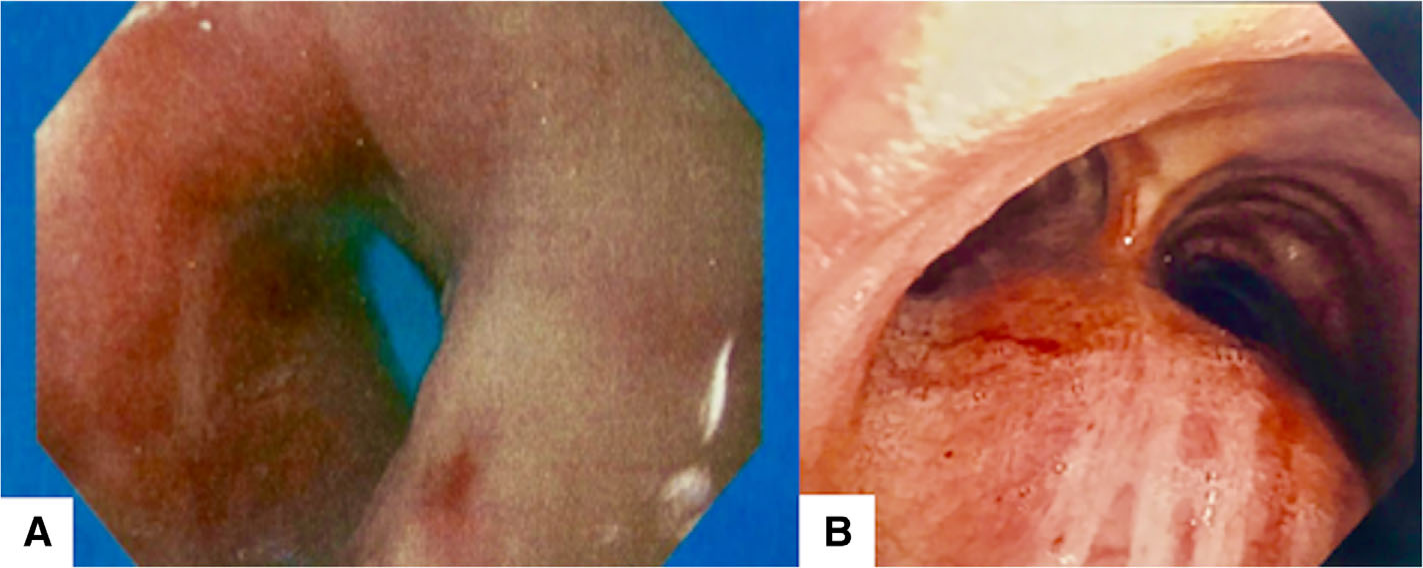
Video‐endoscopic images. (A) Stenosis of the oesophageal orifice. (B) Bronchoscopic inspection reveals external compression of pars membranacea in the caudal part of trachea.

**Figure 2:**
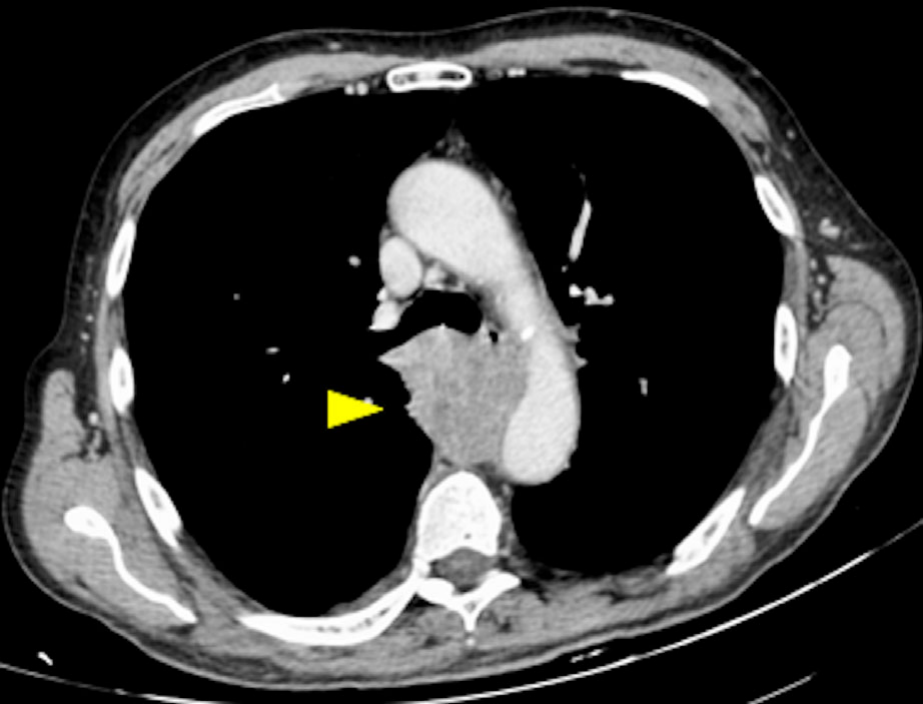
Chest computed tomography (CT) scan. Mediastinal window image shows a solid lesion in the posterior mediastinum (yellow arrow head).

**Figure 3:**
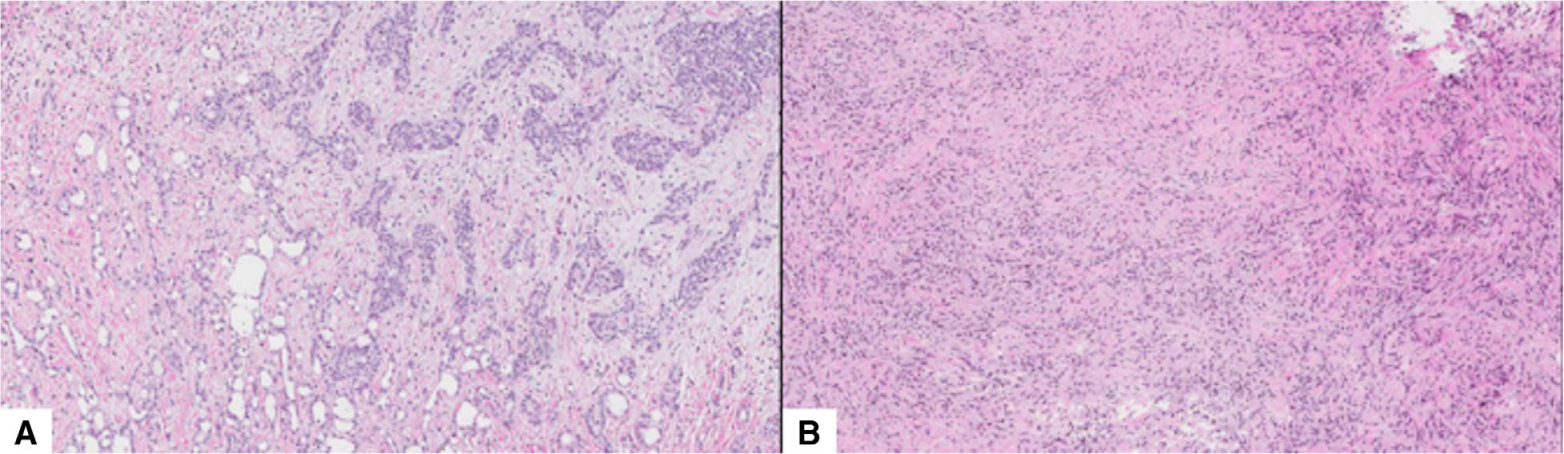
Anatomopathological findings. (A) Proliferation of epithelioid mesothelial cells with round nuclei and small nucleoli, organized in tubules with adenomatoid‐like areas. CK 5/6, calretinin, vimentin, and podoplanin positivity. (B) Proliferation of spindle cells with elongated nuclei. p53, vimentin and focal calretinin positivity (8×, haematoxylin and eosin (H & E) stain).

A 73‐year‐old man was admitted to our hospital having progressive difficulty in swallowing solid foods and persistent cough. He was not a smoker and had no past medical history of cancer or infectious disease. However, the patient reported weight loss and a long‐term exposure to asbestos as a worker in a steel‐making industry. Oesophagogastroscopy showed a narrowing of the distal oesophagus (Fig. 1A) due to extrinsic compression but no evidence of local malignancy. Contrast‐enhanced chest computed tomography (CT) revealed a posterior mediastinal mass, measuring 51 × 43 mm (Fig. 2). As cytological examination of the mediastinal lesion, after bronchoscopy‐guided transtracheal fine‐needle aspiration biopsy (Fig. 1B), was inconclusive and the patient was not considered an ideal candidate for percutaneous image‐guided core needle biopsy, due to uncontrollable cough, a diagnostic right thoracoscopy for surgical biopsies of the mass was then performed. Histopathological examination of the specimens revealed biphasic malignant pleural mesothelioma (Fig. 3). Dysphagia is an unusual form of presentation of malignant mesothelioma and the posterior mediastinum is an uncommon site for this pleural tumour.

### Disclosure Statement

Appropriate written informed consent was obtained for publication of this case report and accompanying images.

